# Scalable Production of Metal Oxide Nanoparticles for Optoelectronics Applications

**DOI:** 10.1002/chem.202401711

**Published:** 2024-12-11

**Authors:** Luca Rebecchi, Irene Martin, Ivet Maqueira Albo, Priyadarshi Ranjan, Teresa Gatti, Francesco Scotognella, Andrea Rubino, Ilka Kriegel

**Affiliations:** ^1^ Functional Nanosystems Istituto Italiano di Tecnologia via Morego 30 16163 Genova Italy; ^2^ Dipartimento di Chimica e Chimica Industriale Università degli Studi di Genova Via Dodecaneso 31 16146 Genova Italy; ^3^ Department of Applied Science and Technology Politecnico di Torino Corso Duca degli Abruzzi 34 10129 Turin Italy; ^4^ Dipartimento di Fisica Università Degli Studi di Genova Via Dodecaneso 33 16146 Genova Italy

**Keywords:** Scale up, Nanocrystals, Metal Oxides, Optimization, Optoelectronics

## Abstract

This work describes the scalability process of a continuous‐injection protocol employed to produce tin‐doped indium oxide nanocrystal dispersions. Different levels of manipulation starting from the synthesis and processing also related to the tuning of the optical response (considering the peculiar combination of UV and NIR absorption with visible transparency) make these materials incredibly versatile. But one of the most attractive features concern the modulation of their charge carrier density through chemical or post‐synthetic doping, as for the case of core‐shell materials, expanding the properties of the core composition. In addition, the colloidal nature of such materials allows for easy solution processing which enables an extensive use in different applications within current thin films base technologies. It is therefore important to push forward the lab‐scale synthesis to properly address the commercial fabrication requirements without any loss in quality. Uniformity is crucial for industrial applications, ensuring predictable performance and facilitating the integration of these nanoparticles into optoelectronic devices. The method here developed allowed a transition from mg‐scale to gram‐scale product mass outputs, while retaining stability in terms of particle size distribution, morphology, crystallinity, and optical properties. This research establishes a robust framework for the scalable production of metal oxide nanoparticles with consistent properties, enhancing their viability for widespread use in optoelectronic applications.

## Graphical abstract

The scaling‐up process of a continuous injection protocol used to produce tin‐doped indium oxide nanocrystal dispersions is described. Synthesis and processing were controlled with special attention to increasing synthesis yield and controlling product quality.

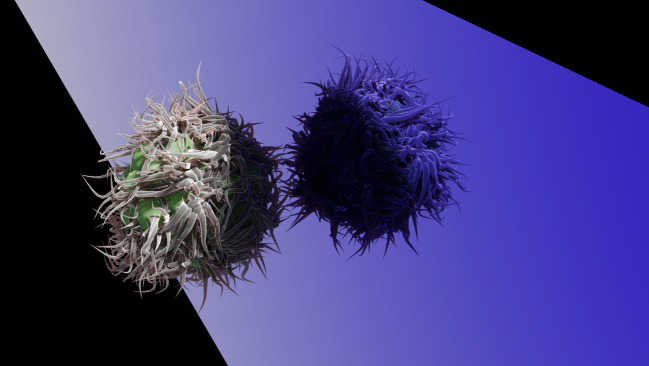

## Introduction

Metal oxide nanocrystals (MO NCs) represent a fundamental class of materials in nanotechnology, characterized by unique and versatile physico‐chemical properties.^[1]^ These nanocrystals, typically composed of metal elements such as indium,[^2^, ^3^] zinc,[^4^, ^5^, ^6^] titanium,[^7^, ^8^] or iron[^9^, ^10^] with oxygen, exhibit exceptional stability,^[11]^ high surface‐to‐volume ratios, and tunable optical and electronic properties.[^12^, ^13^] Scientifically, their controlled synthesis and manipulation at the nanoscale have enabled breakthroughs in various fields, including catalysis,^[14]^ sensing,^[15]^ energy conversion,[^16^, ^17^] and biomedical imaging.^[18]^ Moreover, their use in high‐performance energy storage devices, such as lithium‐ion batteries and supercapacitors,[^19^, ^20^] has generated advancements in sustainable energy technologies. MO NCs have fostered the development of more efficient sensors for environmental monitoring, enabling real‐time detection and remediation of pollutants.^[21]^ Furthermore, their integration in biomedical applications, like targeted drug delivery and imaging,[^18^, ^22^] promoted enhanced therapeutics and precise disease diagnosis.^[23]^

Typically, the most popular synthetic procedures employed for such nanomaterials range from hydrolysis/alcoholysis of inorganic precursors yielding oxides precipitation[^24^, ^25^, ^26^] to thermal decomposition and esterification of intermediates generated with metal‐organic precursors.[^2^, ^27^, ^28^] However, these protocols rarely illustrate syntheses producing over a gram of material,[^24^, ^25^, ^26^, ^29^, ^30^] and in several cases, require specialized equipment[^31^, ^32^] such as microwave reactors^[30]^ or ovens capable of annealing at very high temperatures.^[33]^

Among MOs NCs, recently Indium Tin Oxide (ITO) nanocrystals have been studied extensively for their optoelectronic properties[^2^, ^34^] and their ability to interact with light in the UV and NIR range. Indeed, these nanomaterials have their bandgap in the UV, and possess a tunable localized surface plasmon resonance (LSPR) in the near infrared.[^35^, ^36^] This tunability of the LSPR peak is linked to factors such as free charge carrier density and change in dielectric environment. This last parameter can be related to a variety of factors, among which are the use of different ligands and different solvents in which the particles may be dispersed. ITO NCs’ optical and electronic properties can be modified by controlling shape, dimensions, and composition[^37^, ^38^] as well. Finally, ITO NCs have also shown the ability to store charges upon illumination, prompting investigations on their exploitation for energy storage.[^28^, ^39^, ^40^, ^41^, ^42^, ^43^] Indeed, by illuminating the NCs with light beyond the bandgap, extra electrons are accumulated in the material. This change in free charge carriers density can be monitored spectroscopically with a blueshift in position and increase in intensity of the LSPR peak (as the material charges), and titration of the accumulated charges.^[44]^ Moreover previous reports unveiled the enhancement of such photo‐response when engineering the chemical composition and the depletion region with specifically design core‐shell architectures.[^3^, ^43^]

One of the key advantages of ITO nanocrystals over monolithic ITO films, which are typically fabricated using costly techniques like sputtering or vapor deposition, is the potential for lower‐cost, scalable production through colloidal synthesis combined with solution processing. This approach not only reduces manufacturing expenses but also allows for greater flexibility in substrate selection, making ITO NCs suitable for incorporation into flexible or polymeric matrices. It has been demonstrated already that a resistivity as low as 133 Ω/ϒ can be reached,^[45]^ by solution processing: these values can become competitive with commercially available, traditionally deposited ITO NCs,^[46]^ as they possess a resistivity just one order of magnitude lower. Finally, the use of ITO NCs could allow the control over structural film parameters such as porosity and nanoscale order, allowing for more complex architectures as already demonstrated with other classes of materials.^[16]^ A possible challenge for the large‐scale use of such a material could be the ligands capping the NCs. Usually of organic nature, they require specific protocols to be eliminated from the surface, to result in a high quality, higher conductivity thin film. To this end, literature work points to several approaches, from chemical ligands etching^[47]^ to thermal treatment.^[2]^

So far, most reported synthesis protocols tuning these fundamental parameters have focused only on small scale[^24^, ^25^, ^26^, ^29^, ^30^] syntheses.

In this work, we focus on redesigning the versatile protocol published by Ito et al.,^[48]^ which enables the growth of dimensionally and chemically controlled ITO NCs. We scaled up the protocol's product mass by two orders of magnitude. Moreover, we maintained the morphological, structural, and optical quality of the produced NCs across the different levels of the upscaling process. The control established on the NCs’ size and optoelectronic response confirm the possibility of ITO NCs large‐scale synthesis, paving the way for future industrial applications.

## Materials and Methods

### Standard Indium Tin Oxide and Indium Tin Oxide/Indium Oxide Nanocrystals Synthesis

Indium tin oxide (ITO) nanocrystals (NCs) were synthesized using a continuous injection technique, as reported in literature.[^27^, ^49^] The protocol resembles the living polymerization process, where the reaction progresses through the constant introduction of monomers and stops when no more monomers are added. Similarly, this approach involves injecting metal‐organic precursors to precisely regulate the chemical makeup and particle size, achieving nanometre‐scale control.^[27]^ A brief overview of the procedure for conducting a standard, small‐scale synthesis is provided next.

Tin (IV) acetate (CAS: 2800–96‐6), indium (III) acetate (CAS: 25114–58‐3), oleyl alcohol (technical grade, 85 % purity, CAS: 143–28‐2), and oleic acid (technical grade, 90 % purity, CAS: 112–80‐1) were sourced from Sigma‐Aldrich. Initially, 13 mL of oleyl alcohol was placed in a 100 mL three‐neck round‐bottom flask and heated to 150 °C for 3 h under stirring, with nitrogen gas flow for degassing. The final chemical composition of the nanocrystals (NCs) can be tailored by altering the molar ratio of indium to tin precursors, thereby closely tuning the properties of the synthesized material. A combined total of 1 mmol of indium and tin precursors and 2 mL of oleic acid were introduced into a 50 mL three‐neck, round‐bottom flask. This setup was then heated to 150 °C under a nitrogen atmosphere for degassing using a Schlenk line, facilitating the formation of tin and indium oleates through the reaction of metal acetates with oleic acid.

The reaction produces metal oleates, which serve as precursors for the nanocrystals (NCs), and acetic acid, released from the solution as gas and purged with the continuous flow of inert gas. The mixture is continuously stirred and kept at temperature for three hours to ensure the completion of the reaction. Subsequently, the flask containing oleyl alcohol, used as the reaction medium, was kept under a nitrogen flow of 0.130 L/min and heated to 290 °C. To enhance heat retention and ensure even heating, the glass parts of the flask were insulated with aluminium foil. Then, the metal oleates were cooled to 75 °C before being transferred into a purged polypropylene syringe. The temperature was lowered to prevent the syringe from melting. The syringe was purged by filling it with inert gas from the precursors’ flask and then evacuating the gas out of it three times. Finally, the precursors were injected into the heated oleyl alcohol at a 0.3 mL/min rate using a syringe pump. In the flask, metal oleates transform into metal hydroxides (and subsequently oxides) via esterification with oleyl alcohol to yield oleyl oleate and water vapor as byproducts. This vapor is expelled from the flask by the continuous flow of nitrogen. Oleyl oleate is separated after synthesis by centrifuging the particles and discarding the liquid above the settled precipitates. Around 15 min after the injection, the heat source was removed, and the solution was rapidly cooled using a stream of compressed air to spray acetone over the flask. When the temperature reached approximately 160 °C, the air was stopped, and an ice‐water bath was introduced for even faster cooling (given that the glassware can tolerate a maximum temperature difference of 160 °C, the initial cooling phase cannot employ a water bath). The solution was then centrifuged at 5540 G for 8 min, repeated twice, with ethanol used as an antisolvent to precipitate the NCs. The resulting NCs were stored in octane for subsequent use. Completing the entire process requires about five hours for an experienced operator.

The synthesis of ITO/Indium Oxide (IO) core/shell NCs followed a similar protocol as for the ITO NCs synthesis. For a typical small‐scale synthesis, the standard ITO NCs preparation is modified with the addition of a third flask, used for the shell precursors. Briefly, in addition to the ITO procedure, a 50 mL 3‐necked round bottom flask is prepared with 291 mg of Indium (III) acetate and 2 mL of oleic acid, which then undergoes the same treatment as the ITO precursors. To prepare the core/shell NCs, the procedure of ITO NCs synthesis is followed. After the addition of the ITO cores precursors and the NCs growth for 15 min, the precursors for the shell are injected using the same parameters. Following the second injection and a further 15‐min growth period for the shell, the reaction is quenched as for the ITO NCs case. The purification process remains unchanged.

### Reaction Yield Computation

The yield was determined by calculating the ratio between moles of ITO produced and the moles of precursors used. The molar mass of ITO samples varied slightly in composition and was estimated based on the composition derived from ICP‐OES analysis. The molar yield of ITO was calculated (as outlined in Equation 1) by multiplying the mass yield by the ITO molar mass, and both values were obtained through ICP‐OES.

(1) $$Reaction\;yield\;\left(\%\right)=2^{*}\;\frac{ITO\;mol}{precursors\;mol}=2^{*}\;{\frac{ITO\;NCs\;molar\;mass^{*}ITO\;NCs\;mass\;yield\;}{precursors\;mol}}^{*}100$$

### Larger Indium Tin Oxide Nanocrystals Syntheses

To obtain larger batches of ITO NCs, the standard procedure described above was scaled up several times. The scale‐up was accomplished by beginning with the standard synthesis protocol and doubling the quantity of precursors with each iteration (refer to Table 1 for details). Three‐necked round‐bottom flasks were used. We selected the size of these vessels based on the required volume, never filling the flask more than halfway (for example, a 500 mL flask would contain no more than 250 mL). This approach was taken to guarantee uniform heating of the solution, considering that the heating mantles only cover the flasks’ lower half.

**Table 1:** The table presents the experimental injection rates and quantities of precursors used in scaling up the synthesis of ITO NCs. The injection rate and the amounts of precursors increase proportionally with the synthesis scale.

**Total precursors** **(mmol)**	**Indium (III)** **acetate (mg)**	**Tin (IV)** **acetate (mg)**	**Oleyl alcohol** **(mL)**	**Oleic acid** **(mL)**	**Precursor injection** **rate (mL/min)**
1	263	45	13	2	0.3
2	526	90	26	4	0.6
4	1052	180	52	8	1.2
8	2104	360	104	16	2.4
16	4208	720	208	32	4.8
32	8416	1440	416	64	9.6

To keep the reaction conditions as similar as possible to those of the standard synthesis, the rate at which precursors were injected into the reaction mixture was scaled up in direct proportion to the increase in the mass of the precursors. This adjustment ranged from an initial rate of 0.3 mL/min to 9.6 mL/min at the largest scale (Table 1). Reaction temperatures, times for preparing the oleates, and the nucleation and growth phases of the nanocrystals were maintained at the same values as those used in the standard synthesis process.

### Ligand Exchange

Ligand exchange is achieved with the following procedure: a vial we prepared 5 mL of a 20 mg/mL ITO NCs solution in hexane. Then, we added 10 mL of a 20 mM Tetrabutylammonium hydroxide (TBAOH) solution in ethanol. If the colloidal solution is uniform, the ligand exchange is undergoing. We shook the vial vigorously for 10 min with a lab shaker. Cleaning steps involved adding 30 mL of toluene to the mixture and transferring it to Falcon tubes, which were centrifuged at 7512 G for 8 min. The supernatant was discarded, and the precipitate was redispersed in 5 mL of TBAOH 20 mM stock solution. Sonication helped in redispersion. We then added 15 mL of toluene, and recentrifuged with the same parameters. Finally, the precipitate was collected and dispersed in 5 mL TBAOH 20 mM stock solution for storage.

### Materials Characterization

Optical characterization of NCs was carried out with an Agilent Cary 5000, using an optical cuvette made of Infrasil®, with an optical path of 2 mm (Starna Scientific). First, the spectrum of blank solvent was acquired, then a second spectrum was acquired upon introducing the sample in the cuvette. Finally, to obtain the spectra presented in this paper the solvent spectrum is subtracted from the one containing sample and solvent. The area in which interferences from the solvent absorption features are too intense, are then masked. Clean solvent spectra are provided in Figure S6.

The chemical composition and concentration of NCs solutions were calculated using inductively coupled plasma optical emission spectroscopy (ICP‐OES). Concentration values computed using ICP‐OES only account for the inorganic mass of the ITO NCs.

Transmission Electron Microscopy (TEM, JEOL JEM‐1400Plus‐Analytical 120 kV TEM/STEM) was employed to characterize dimensions and morphology. Image analysis was performed using ImageJ software (Version 1.51).

X‐Ray Diffraction (XRD) patterns were acquired by dropcasting NCs solutions on a 0‐diffraction silicon substrate, then letting the solvent evaporate. Measurements were performed using a PANanalytical Empyrean X‐ray diffractometer, operating at 45 kV and 40 mA, and a 1.8 kW Cu Kα X‐ray tube. XRD patterns were acquired in ambient conditions, working with a parallel beam geometry with symmetric reflection mode. Analysis of patterns was performed using XRD2DScan Software (version 6.00.0001) to convert from 2D to 1D patterns, and the HighScore Software (version 4.9.0) to find, match, and identify diffraction peaks to establish the crystalline structure of the analyte. Finally, Origin 2020 (version 9.70.188) was used to plot the patterns along with their matching reference pattern card.

## Results and Discussion

**Figure 1:**
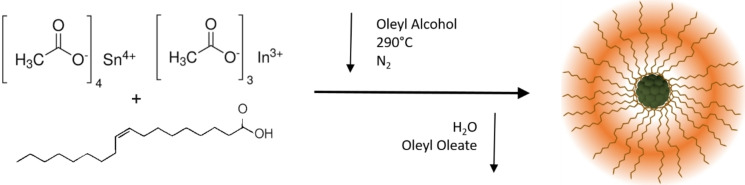
Illustrated is the condensation mechanism inducing nucleation and growth for ITO NCs. The metal oleates react with the oleyl alcohol, yielding metal oxides while producing water steam and oleyl oleate as byproducts.

The ligand‐assisted colloidal synthesis employed for the nanoparticle's preparation is illustrated in Figure 1, which describes the reaction mechanism for the nucleation and growth of ITO NCs. The method consists in the preparation of indium and tin oleates, which then undergo esterification with oleyl alcohol, forming metal hydroxy species that are then dehydrated in metal oxide, producing water and oleyl oleate as byproducts. Once the nucleation phase has been completed, NCs growth is governed by controlling the injection rate of precursors, yielding uniform particles with a narrow size distribution.

The outlined synthesis process is quite labour‐intensive, with respect to simpler methods, like a heat‐up or a hot injection. Nonetheless, considering the published literature,^[48]^ the heat‐up method was demonstrated to be inadequate for controlling NCs size, while hot injection led to rapid water vapor formation, adversely affecting the synthesis.^[48]^ Given these factors, the slow injection method described previously was selected for its superior results.

### Mass Yield and Reaction Yield

Experimental work started by reproducing the published protocols for ITO NCs,^[48]^ employing a total of 1 mmol of metal precursors. This amount was taken as the starting point for our work. Furthermore, the optical, structural, and morphological properties of the NCs produced with 1 mmol of precursors were used as a reference to compare with the subsequent, larger‐scale batches. The reported procedure was reproduced several times at the 1 mmol scale (see Table 1 for further details) to improve proficiency and reduce operator‐related errors. Once a good command was acquired during the synthesis, we increased reagent quantities progressively (exponentially) (see Table 1). More specifically, six different batch scales have been prepared, from A to F, using 1 mmol, 2 mmol, 4 mmol, 8 mmol, 16 mmol, and 32 mmol of precursors, respectively.

**Figure 2:**
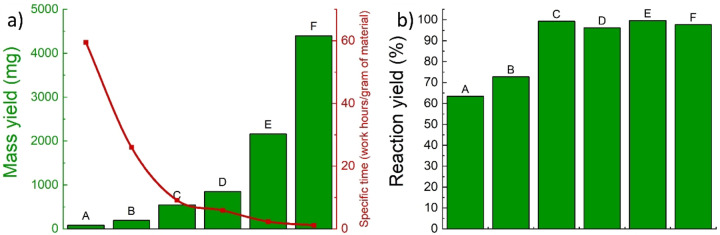
a) The green histogram shows a significant increase in product mass as the synthesis scale grows exponentially. The red line tracks the decrease in specific time–measured in work hours per gram of product–as the system is scaled up, assuming a constant effort of 5 work hours for each batch, independent of its size. b) Reaction yield % (mol/mol) across various synthesis scales demonstrates an initial increase, stabilizing at nearly 100 % from the 4 mmol batch size onwards. This stabilization is attributed to the diminished impact of mechanical losses as the scale increases. For exact mass yield figures, please see Table 2.

In Figure 2a, we illustrate how the mass of the product (highlighted in green, calculated by considering only the inorganic component of the nanocrystals) increases significantly–by two orders of magnitude–as the batch size grows. For the largest batches, we reached a multi‐gram scale, improving the total mass yield with respect to the published synthetic protocols. For precise mass values, refer to Table 2.

**Table 2:** The table lists dimensional data and mass yields relative to different scales ITO NCs syntheses. The distribution of NC sizes remains consistent within the same batch, with variations of only a few percentage points and no discernible trend as the batch size increase from A to F. Considering instead the same type of batch, as in the case of D‐sized syntheses, the diameter variability increases up to +/−2–3 nm (with an average diameter of 13.5 nm, computed from Tables 2, S2, and S3). Additionally, the table reports a reduction in the specific time required (hours per gram of produced material).

**Precursors used** **(mmol)**	**Diameter** **(nm)**	**% uncertainty** **diameter**	**Product mass** **(mg)**	**Specific time** **(work hours/gram** **of material)**
A (1)	7.4 +/−0.8	+/−10.8 %	84	59.5
B (2)	7.8 +/−1.2	+/−15.3 %	192	26.0
C (4)	10.9 +/−1.5	+/−13.8 %	547	9.1
D (8)	12.7 +/−1.8	+/−14.2 %	850	5.9
E (16)	12.9 +/−1.9	+/−14.7 %	2161	2.3
F (32)	12.7 +/−1.8	+/−14.2 %	4398	1.1

Additionally, we evaluated the advantages of scaling up the synthesis regarding time efficiency. By applying Equation (2), we calculated the specific time required to produce 1 gram of ITO nanocrystals. Given that an expert operator can complete the synthesis in five work hours for batches of any size, the specific production time significantly reduces from approximately 59 h to about 1 work hour per gram of material. This efficiency gain across different process scales is represented with the red line in Figure 2a.

(2) $$Specific\;time\;\left(\frac{hours}{g}\right)=\frac{5\;hours\;}{mass\;yield\;\left(g\right)}$$

After determining the absolute mass yield via ICP‐OES analysis, we calculated the reaction's molar yield in %, using Equation (1). This step evaluated how scaling up the process affected the molar yield and to what extent. Illustrated by the histogram in Figure 2b, the reaction yield (in %) improves consistently across different synthesis scales, ranging from about 60 % for the smallest batch to nearly 100 % for larger batch sizes. Generally, lower yields are attributed to operator mistakes and mechanical losses (for example, material losses during purification or residues left on glassware), which are relatively consistent across all batch sizes. However, these factors significantly impact smaller batches, whereas their influence diminishes with larger scale, underscoring the advantages of scaling up the synthesis process.

### Morphological Analysis

After achieving large‐scale production of NCs, we undertook their characterization to evaluate the extent of control maintained over the material's properties during the scaling‐up process, comparing it to the smaller, standard‐sized synthesis.

**Figure 3:**
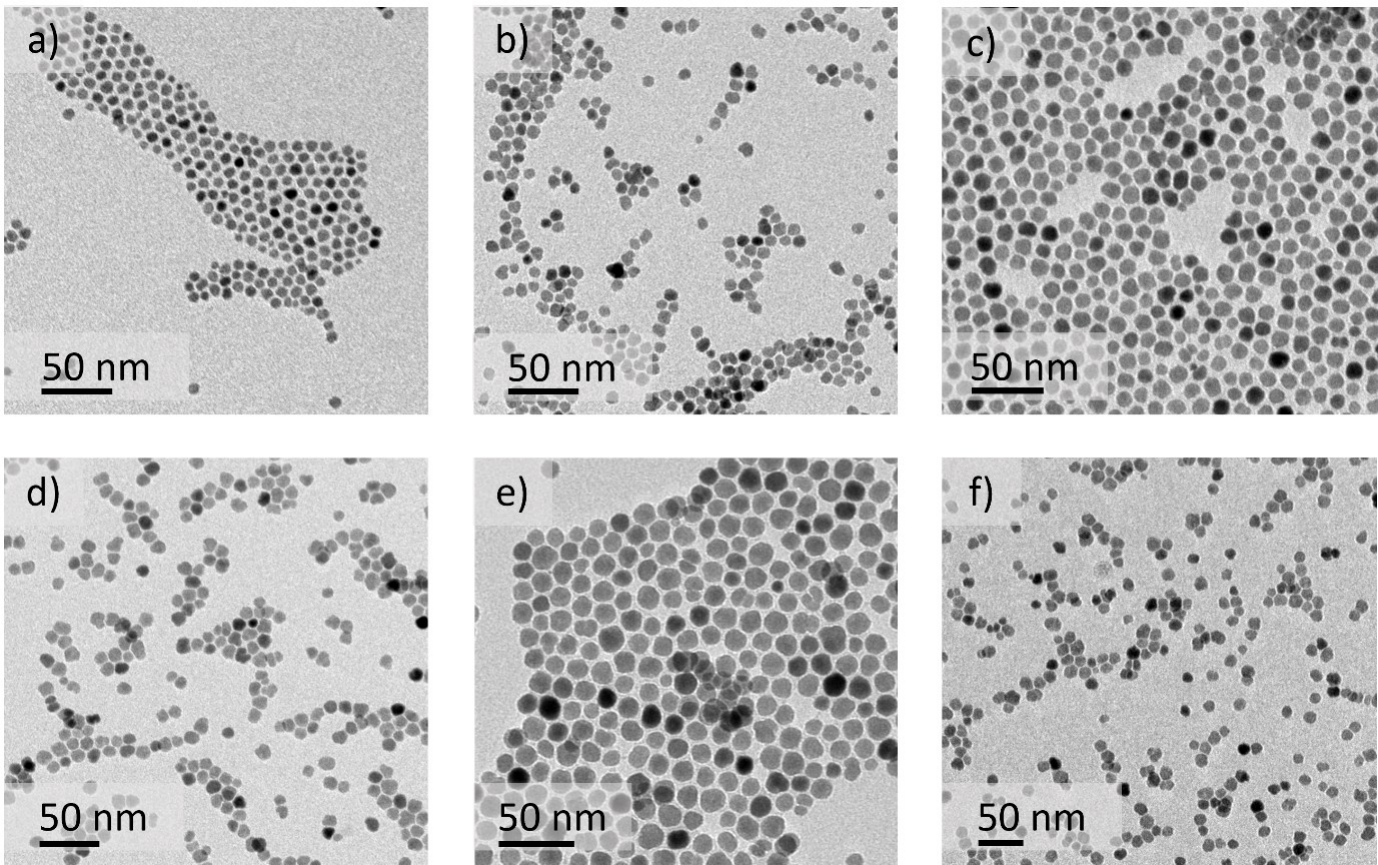
From panels a) to f), we show ITO NCs produced using 1 to 32 mmol of metal precursors. In Table 2, we report the evolution of particle size and size distribution across different sample sizes. As indicated in Table 2, the uncertainty in particle size consistently stays within a range of a maximum of +/−15 % within the same batch.

Among other things, ITO NCs’ optoelectronic properties depend on NCs doping and size distribution. To quantify how size distribution varies across different batches, we characterized them using TEM imaging. Subsequently, we measured the NCs’ size with image analysis and extrapolated a particle size distribution. In Figure 3, we show representative TEM images of the NCs produced for batches from A to F (as highlighted earlier), respectively, from panels a) to f). Each sample displays NCs with pseudospherical shape and a controlled, monodisperse size distribution. In Figure S1, in the Supporting Information (orange bin columns), we show the diameter distribution of all batches, closely following the applied Gaussian fitting (black, continuous curve). The average diameters achieved are in the order of 10 nm, with a size distribution within a +/−1–2 nm range, within the same batch. Considering instead the same type of batch, as in the case of batch D, the diameter variability increases up to +/−2–3 nm (diameter average of 13.5 nm, computed from data in Tables 2, S2, and S3).

**Figure 4:**
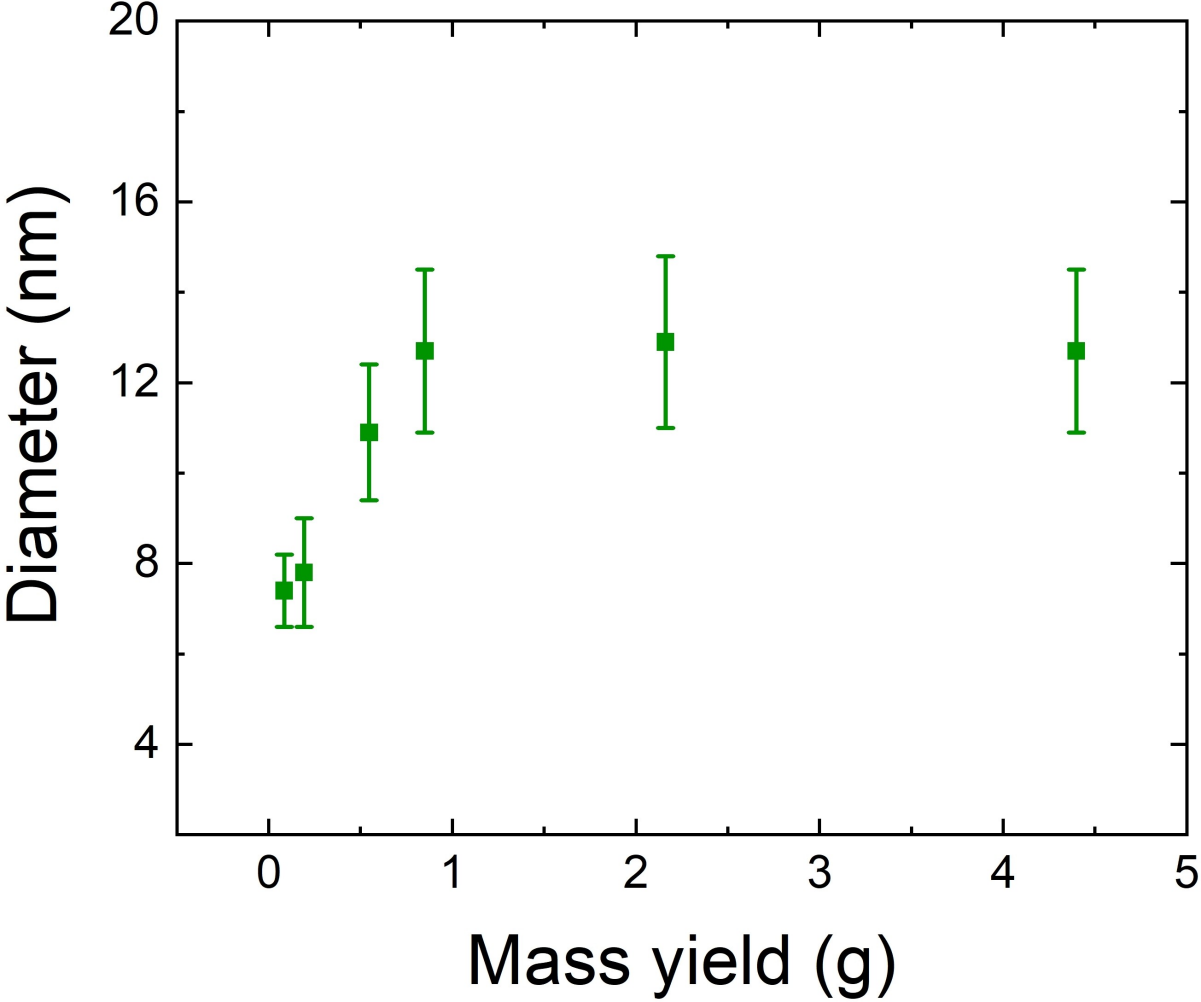
Evolution of the diameter as a function of synthesis batch size. The diameter can be seen increasing with batch sizes, maintaining the other synthesis conditions constant.

In Table 2, we collect the results of our analysis. The size distribution, expressed as a percentage, oscillates between +/−11 and 15 %, thus not varying notably within the same batch. However, this variability increases when considering same sized inter‐batch differences, as seen in the previously mentioned case of D‐sized batches, where the size distribution reaches up to 22 %. Moreover, as shown in Figure 4 (and detailed in Table 2), when comparing the two extremes in synthesis batch size, we observed that the average particle diameter is larger in the largest synthesis batches compared to the smallest, with all other parameters held constant. To investigate the reason, at first, we hypothesized that larger synthesis volumes experienced slower cooling rates, and thus longer growth times. To test it, we performed two additional experiments on different scales, D and F. In both cases, we took aliquots just before quenching the reaction. Larger batches exhibited more particle growth (during cooling) as compared to smaller ones (see Table S2 for results). However, the diameter increase during the cooling phase was sub‐nanometric, suggesting a negligible role of cooling on particle size.

We then modified our approach by taking aliquots during NC growth at various scales to monitor growth progression. The results, presented in Table S3 were unexpected. They underscore how, within the same batch, different grow times do not change drastically the particles size. In fact, particle diameters of the same batch remained relatively stable, even when taking aliquots early during the growth period. It was observed that the nanoparticle diameters across batches reached their maximum size between 4 and 7 min of growth. Furthermore, monitoring the NCs dimensions demonstrated a tendency toward size focusing, with diameters stabilizing just below their peak values.

Given our results, the reason for large particle growth in bigger batches remains unclear, underscoring the need for further investigation. Furthermore, the difference in diameter attained among batches of the same size (i. e. same amount of precursors) is still an open question, which requires further experiments and will be addressed in a future work. Automation may help better control NC dimensions by minimizing human error during synthesis.

Lastly, we analysed the crystalline structure of the nanoparticles obtained in the different batches. In Figure S2, we compare the XRD patterns of a 1 mmol scale and 32 mmol scale batches, serving as representative examples of smaller and bigger sizes, respectively. The perfectly overlapping patterns confirm the synthesis reproducibility of the crystalline phase.

### Optical Properties

**Figure 5:**
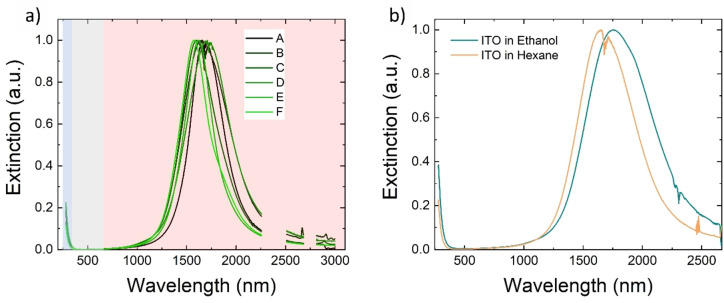
a) Absorption spectra of ITO NCs in a hexane dispersion. In the UV region (light blue area), one can see the absorption onset related to the bandgap of ITO. In the visible range, there is transparency (grey area), and in the NIR, the LSPR peak (light pink area) is located. b) Absorption spectra of ITO NCs dispersed in different solvents: light blue in ethanol, orange in hexane. A shift in the plasmonic peak is present and attributed to the change in the dielectric environment because of the different solvents.

Given the optimal result of the upscaling method, from the morphological point of view, we finally investigated the NCs optical properties. Optical quality has been evaluated by considering the reproducibility of the typical features of ITO NCs in the absorption curve of samples across the scale‐up process. In Figure 5a, we show the absorption spectra for different ITO NC samples of varying batch sizes. All curves show the characteristic Localized Surface Plasmon Resonance (LSPR) peak in the NIR region (shaded in light pink), a transparency window in the visible range (shaded in grey), and, in the UV region, the onset of absorption relative to the material bandgap (shaded in light blue). The LSPR peak position for all samples is found in the 1600–1700 nm range, as typical for highly doped ITO NCs.^[36]^

**Figure 6:**
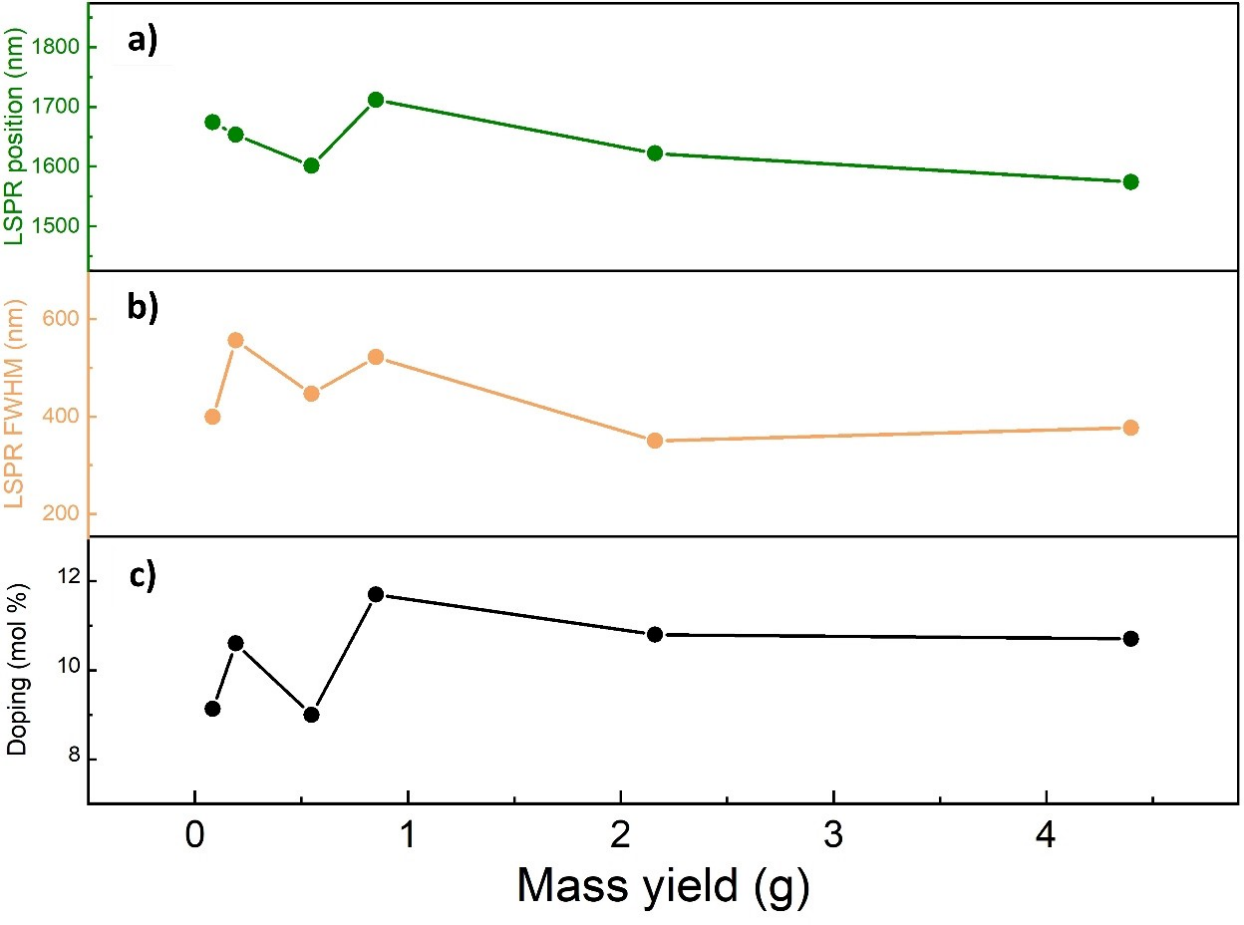
a) The position of the LSPR Peak maximum for various batches (orange curve) is shown, along with b) the Full Width Half Maximum (FWHM) of the corresponding peaks (red curve). In both cases, there is no discernible trend with oscillating composition and batch size, indicating effective control over the optical properties of ITO NCs throughout the scale‐up process. c) Sn doping in different ITO NCs batches, indicating how the composition oscillates around the target composition of 10 % Sn doping.

To better visualize batch‐to‐batch changes in the optical characteristics, we plot in Figure 6 the values of the LSPR peak maximum and the full width half maximum (FWHM) of the same peak. In fact, the FWHM is influenced largely by the particles’ dimensions, shape, and morphology, and it can indicate NCs’ aggregation or size distribution. Meanwhile, the carrier density largely affects the peak position.^[50]^ We observe that with increasing batch sizes, there is no specific trend in terms of spectral position, fluctuating in the range between 1570 and 1720 nm (Figure 6a). As for the case of LSPR peak position, FWHM values do not indicate a specific dependence across different batches (Figure 6b). In Table S1, we report the precise numerical values of FWHM and LSPR peak maximum positions.

Figure 6c depicts the doping concentrations of the different synthesis batches, obtained via ICP‐OES, aiming for a 10 % Sn doping composition target. The values fluctuate between 9 to around 12 %, without a clear trend. This lack of a clear evolution as batch size increases and doping levels oscillate suggests that NCs optical properties remain consistent across different samples, accounting for the intrinsic variability between the batches produced. This hypothesis has been tested (and confirmed) by performing multiple times batches at different sizes. The data, summarized in Table S4, show that while composition may vary between batches, the average results converge toward the target doping level of 10 % +/−1.1 % mol/mol for the biggest batch size. This is a good indication that ITO NCs obtained with a scaled‐up process retain the optical properties (and quality) characteristic of their counterpart coming from small, more controlled batch syntheses.

Such control over the scale‐up synthesis and the scales reached encouraged us to investigate further the use of more interesting solvents for possible following technological steps, like industrial solution processing of the particles. In particular, we used a ligand exchange method to obtain stable colloidal solutions of ITO NCs in polar solvents, like ethanol (shown in Figure S3), which are less toxic than the original hexane and can improve the liquid deposition.^[2]^ Dispersibility is achievable in polar solvents, including MilliQ water, acetonitrile, ethanol, and acetone (as shown in Figure S4). We were able to remove the old oleate ligands using an ammonium compound (tetrabutylammonium hydroxide), which coordinates the NCs surface and stabilizes the particles in solution, replacing the old, covalently bonded ligands. In Figure 5b, we show how the optical properties respond to the change from a non‐polar to a polar solvent. The LSPR peak of ITO NCs slightly red‐shifts, as the NCs in ethanol tend to become more aggregated (see Figure S5 for TEM micrographs), inducing a coupling between the plasmonic resonances of ITO NCs. These results, regarding both control over properties and exchange to less toxic solvents, make the synthetic protocol herein presented a valid step toward industrial scale use in all the different applications already mentioned.

To generalize the method outlined in this work, we examined its scalability and application to other systems. We specifically applied the procedure to create more complex core‐shell structures, where an indium oxide layer is grown around an ITO core. Such a structure has proven to be extremely interesting from the optoelectronic point of view since the properties and typical plasmonic effects of these nanomaterials are sensitive to doping.[^38^, ^49^, ^51^, ^52^, ^53^] The core‐shell architecture built by means of a non‐conductive material coating around the core results in a completely different charge distribution profile in the particles, also owing to the presence of a charge depletion layer.[^35^, ^42^] In our case, we were able to increase the scale of the colloidal synthesis from a A‐sized batch to a C‐sized batch. Upon scaling up the synthesis, both core and core‐shell NCs maintained monodispersity and uniformity. However, similar to the smaller ITO NC cores, the core‐shell particles synthesized in larger batches exhibited increased size compared to those from smaller syntheses (see dimensional analysis in Table S5 and TEM micrographs in Figure S7). The increase in overall particle diameter mainly concerns the core (from about 9 to about 15.5 nm) while the thickening of the shell is less pronounced (from about 3 to about 4.5 nm). In this case, the observed size increase may be attributed to operator‐related reproducibility issues. We can therefore hypothesize that, with sufficient control, the method can also be applied to particles with different shell thicknesses or even different compositions (multi‐shell systems) since shell thickness is more closely linked to the kinetics of the particle coating growth. As the shell grows, the primary LSPR peak may undergo further spectral shifts due to changes in the dielectric environment surrounding the particle. AS reported in literature,^[54]^ such modulation can even lead to the formation of a second absorption peak (see Figure S8), i. e. an even more complex charge density profile that can induce a new resonance separate from the original one and with a behaviour (e. g., the photo‐response) that may even be independent.

## Conclusions

In summary, we have investigated the scalability process of the colloidal synthesis of ITO NCs, comparing the optical, morphological, and structural properties to assess their consistency in different batch sizes. We have shown that increasing the precursors’ mass exponentially allowed to move from milligram to gram scale, dramatically reducing the specific time‐per‐gram of material and increasing the synthesis efficiency by reducing mechanical losses to a negligible term in the bigger batches. Statistical analysis of TEM micrographs indicates that within the same batch, particle size distribution (in %) does not suffer significant changes, although the particle sizes produced do vary from batch to batch. Optical properties have been studied, with LSPR peak position and FWHM varying among batches with no systematic deviations, ascribing this behaviour to the statistical variability from batch to batch. The transferability to polar solvents has been presented as a viable, environmentally friendly alternative to non‐polar media. Finally, scaling up the synthesis of more complex core/shell structures has shown that this approach can be extended to a wider range of materials, offering a potential pathway for scaling up different synthesis protocols through its use.

As further steps, we foresee the use of this protocol on a small batch reactor scale, pushing the production of particles on the kg scale and enabling their use in a broader range of applications, from photochromic windows to energy storage devices.

## Conflict of Interests

The authors declare no conflict of interest.

1

## Supporting information

As a service to our authors and readers, this journal provides supporting information supplied by the authors. Such materials are peer reviewed and may be re‐organized for online delivery, but are not copy‐edited or typeset. Technical support issues arising from supporting information (other than missing files) should be addressed to the authors.

Supporting Information

Supporting Information

## Data Availability

The data that support the findings of this study are available from the corresponding author upon reasonable request.
