# Supplementary material for: Scalable Production of Metal Oxide Nanoparticles for Optoelectronics Applications
Source: Chemistry. 2024 Dec 11;31(8):e202401711. doi: 10.1002/chem.202401711 (PMC11803355; doi:10.1002/chem.202401711)
Supplement: Supplementary file 1 — Supporting Information [file CHEM-31-e202401711-s001.pdf]

Chemistry–A European Journal

Supporting Information

Scalable Production of Metal Oxide Nanoparticles for Optoelectronics Applications

Luca Rebecchi, Irene Martin, Ivet Maqueira Albo, Priyadarshi Ranjan, Teresa Gatti, Francesco Scotognella,* Andrea Rubino,* and Ilka Kriegel*

Supporting Informations

Scalable production of Metal Oxide Nanoparticles for optoelectronics applications

Luca Rebecchi[1,2,3], Irene Martin[1,3], Ivet Maqueira Albo[1,4], Priyadarshi Ranjan[1], Teresa Gatti[3], Francesco Scotognella[3]*, Andrea Rubino[1]*, Ilka Kriegel[1]*

1 Functional Nanosystems, Istituto Italiano di Tecnologia, via Morego 30, 16163 Genova, Italy

2 Dipartimento di Chimica e Chimica Industriale, Università degli Studi di Genova, Via Dodecaneso 31, 16146 Genova, Italy

3 Department of Applied Science and Technology, Politecnico di Torino, Corso Duca degli Abruzzi 34, 10129 Turin Italy

4 Dipartimento di Fisica, Università Degli Studi di Genova, Via Dodecaneso 33, 16146, Genova, Italy

***ilka.kriegel@polito.it, francesco.scotognella@polito.it, andrea.rubino@iit.it**

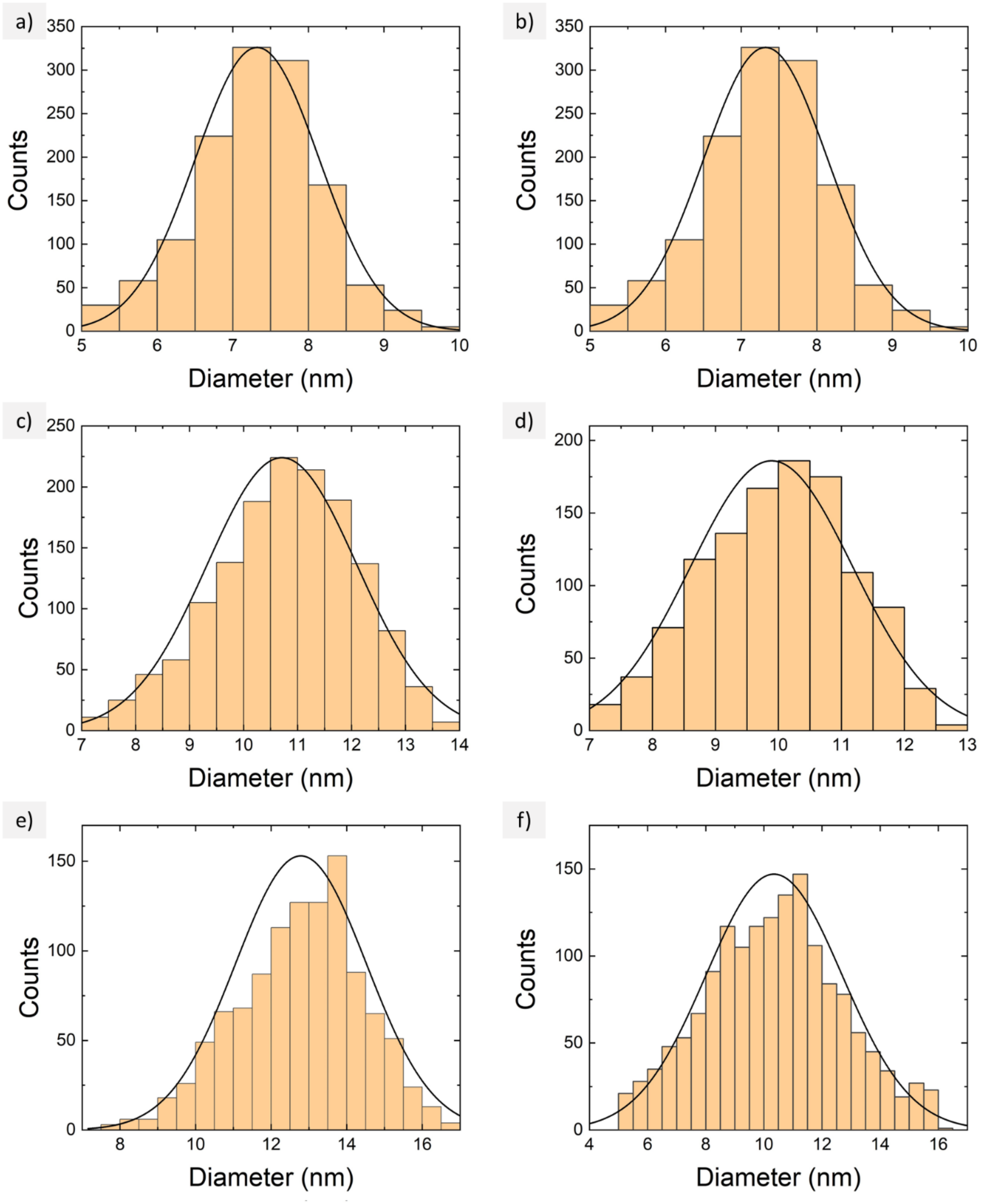

Figure S1 – We show the diameters distribution (orange bin columns), with an applied gaussian fitting (black, continuous curve). From panel a) to f) we show the distribution of the increasingly bigger batches, from 1 mmol to 32 mmol.

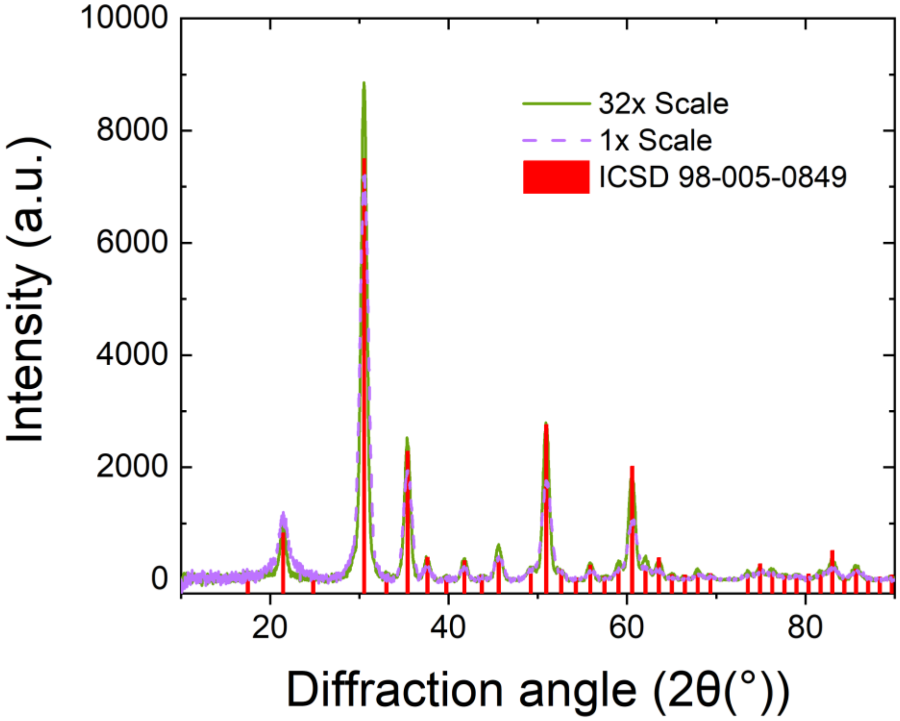

Figure S2 – XRD patterns of the two extremes in the scaling up process. The almost perfect superposition of the diffraction patterns show the formation of the same crystalline structure, with the absence of other phases. Finally, the diffraction patterns are reasonably superimposable with each other.

**Precursors used (mmol)**	**LSPR Peak maximum position**	**FWHM**
1	1674	399 nm
2	1653	556 nm
4	1601	447 nm
8	1712	522 nm
16	1622	350 nm
32	1574	377 nm

Table S1 – FWHM values and LSPR peak maximum position from different sized batches.

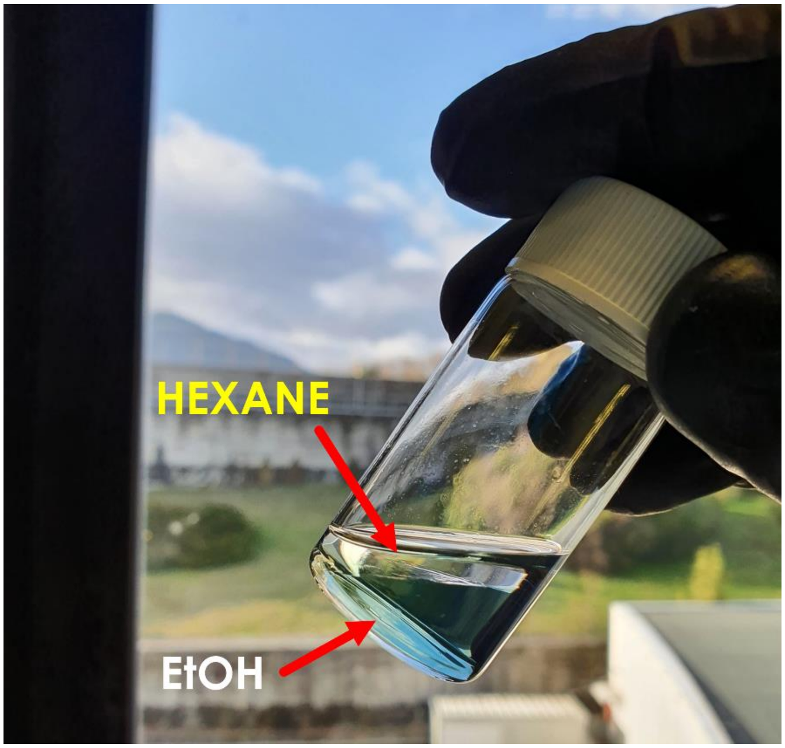

Figure S3 – Represented is the result of the ligand exchange: ITO moves from the top layer of hexane (lighter) to the bottom layer of ethanol (denser), after the procedure is completed. The purification by precipitation of the solution then follows the procedure reported in the Materials and Methods section of the main text.

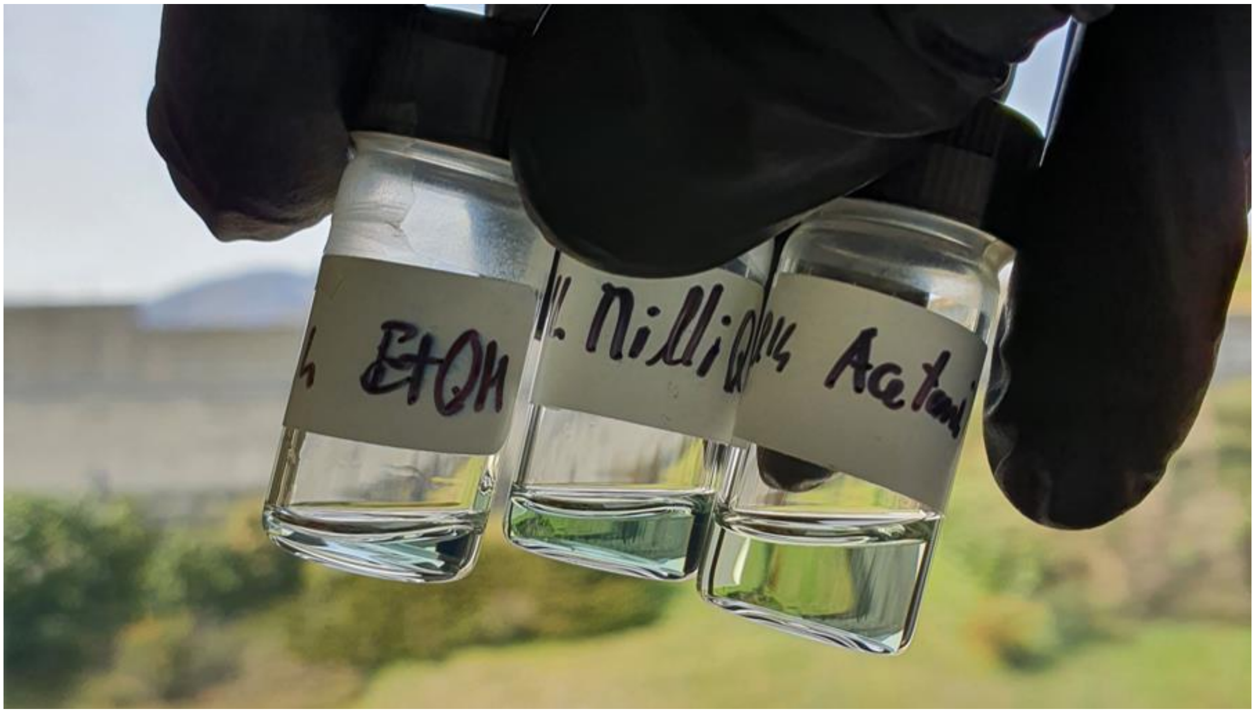

Figure S4 – Diluted ITO colloidal dispersions in different polar solvents, from left to right respectively ethanol, MilliQ water and acetonitrile.

Synthesis scale	Stage	Diameter (nm)
D_2	Aliquot before cooling	11.0 +/- 1.5
D_2	Product after cooling	11.4 +/- 1.6
F_2	Aliquot before cooling	18.3 +/- 2.7
F_2	Product after cooling	18.9 +/- 2.7

Table S2 - Dimensional analysis of particles pre and post quenching - Different synthesis scales. Please note that the here reported ITO NCs batches are different from the ones reported in Table 2.

Synthesis scale	Growth time	Diameter (nm)
C_3	4 minutes	10.3 +/- 1.7
C_3	8 minutes	11.1 +/- 1.5
C_3	13 minutes	10.7 +/- 1.4
C_3	15 minutes	10.9 +/- 1.5
D_3	1 minute	16.4 +/- 2.0
D_3	4 minutes	18.0 +/- 1.7
D_3	7 minutes	17.2 +/- 2.2
D_3	10 minutes	16.8 +/- 2.0
D_3	13 minutes	17.3 +/- 2.4
D_3	15 minutes	16.5 +/- 2.0

Table S3 - Dimensional analysis of aliquots of ITO NCs taken at different times, from a C and D-sized batch. Please note that the here reported ITO NCs batches are different from the ones reported in Table 2.

<table>
<tr><th>Synthesis scale</th><th>Dopant concentration</th><th>Average doping (+/- standard deviation)</th></tr>
<tr><td>A_4</td><td>13.0 %</td><td rowspan="3">9.6 % +/- 1.9 %</td></tr>
<tr><td>A_4</td><td>8.2 %</td></tr>
<tr><td>A_4</td><td>8.9 %</td></tr>
</table>

A_4	9.0 %	
A_4	9.1 %	
F_4	10.7 %	
F_4	8.6 %	9.7 % +/- 1.1 %
F_4	9.9 %	

Table S4 - Doping levels for multiple syntheses at the two extreme batch sizes. Please note that the here reported ITO NCs batches are different from the ones reported in Table 2.

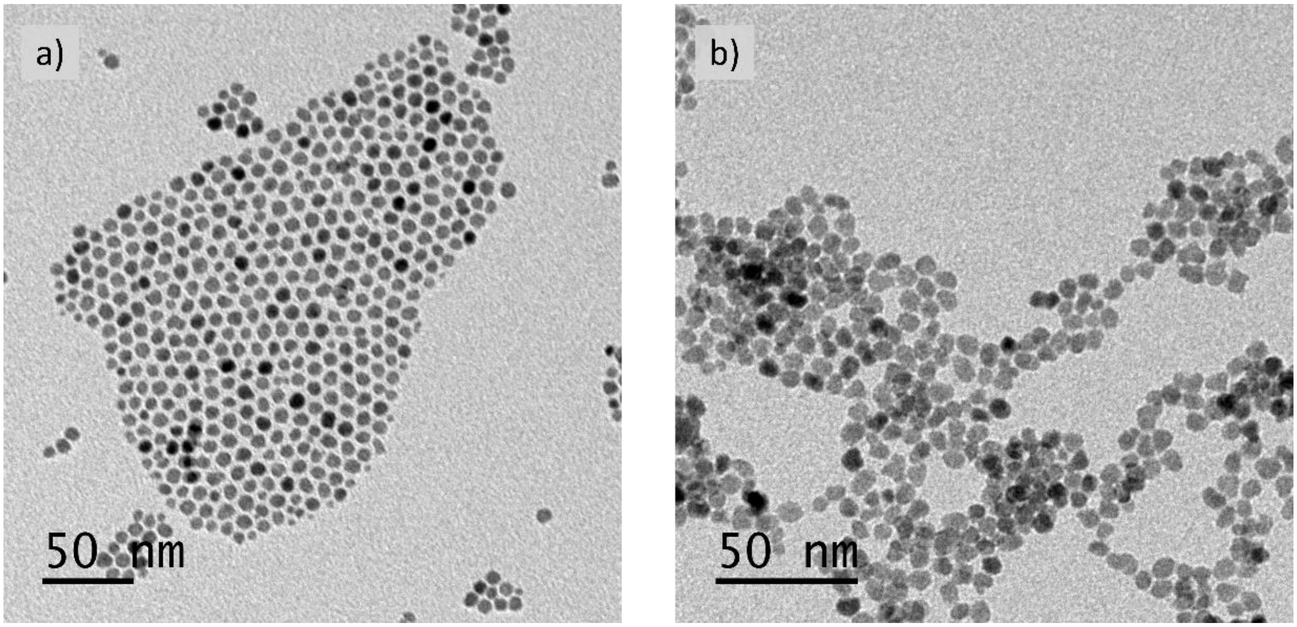

Figure S5 - a) TEM micrograph of ITO NCs dispersed in hexane. b) TEM micrograph dispersed in ethanol

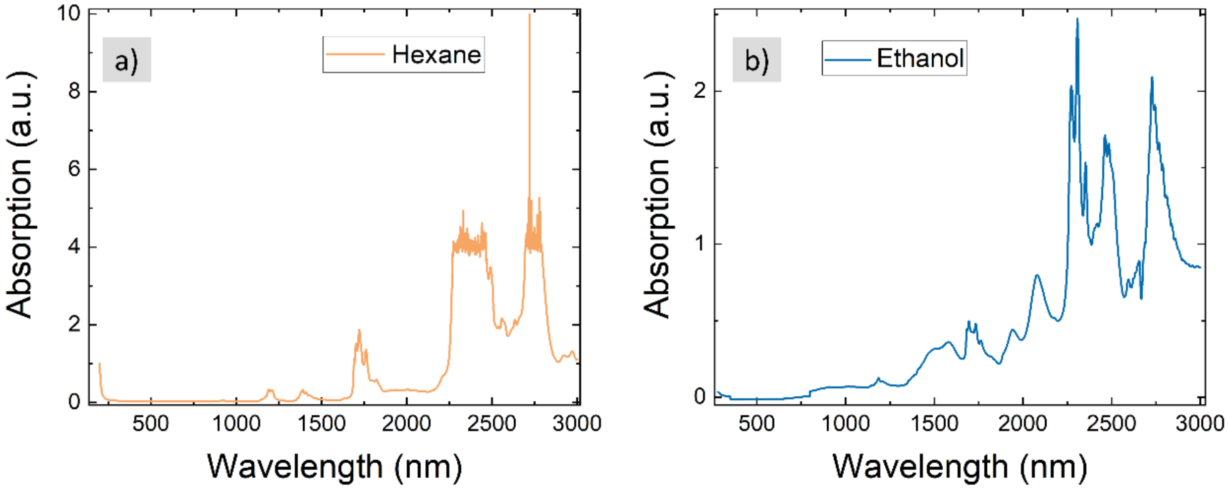

Figure S6 - a) spectrum of pure hexane and b) spectrum of pure ethanol used for spectroscopy

Synthesis scale	Stage growth	Diameter (nm)
A	Core	8.9 +/- 2.0
A	Core/Shell NCs	11.7 +/- 1.1
C	Core	15.4 +/- 2.0
C	Core/Shell NCs	20.1 +/- 1.6

Table S5 - Dimensional analysis of the scaled-up synthesis of the Core/Shell structure

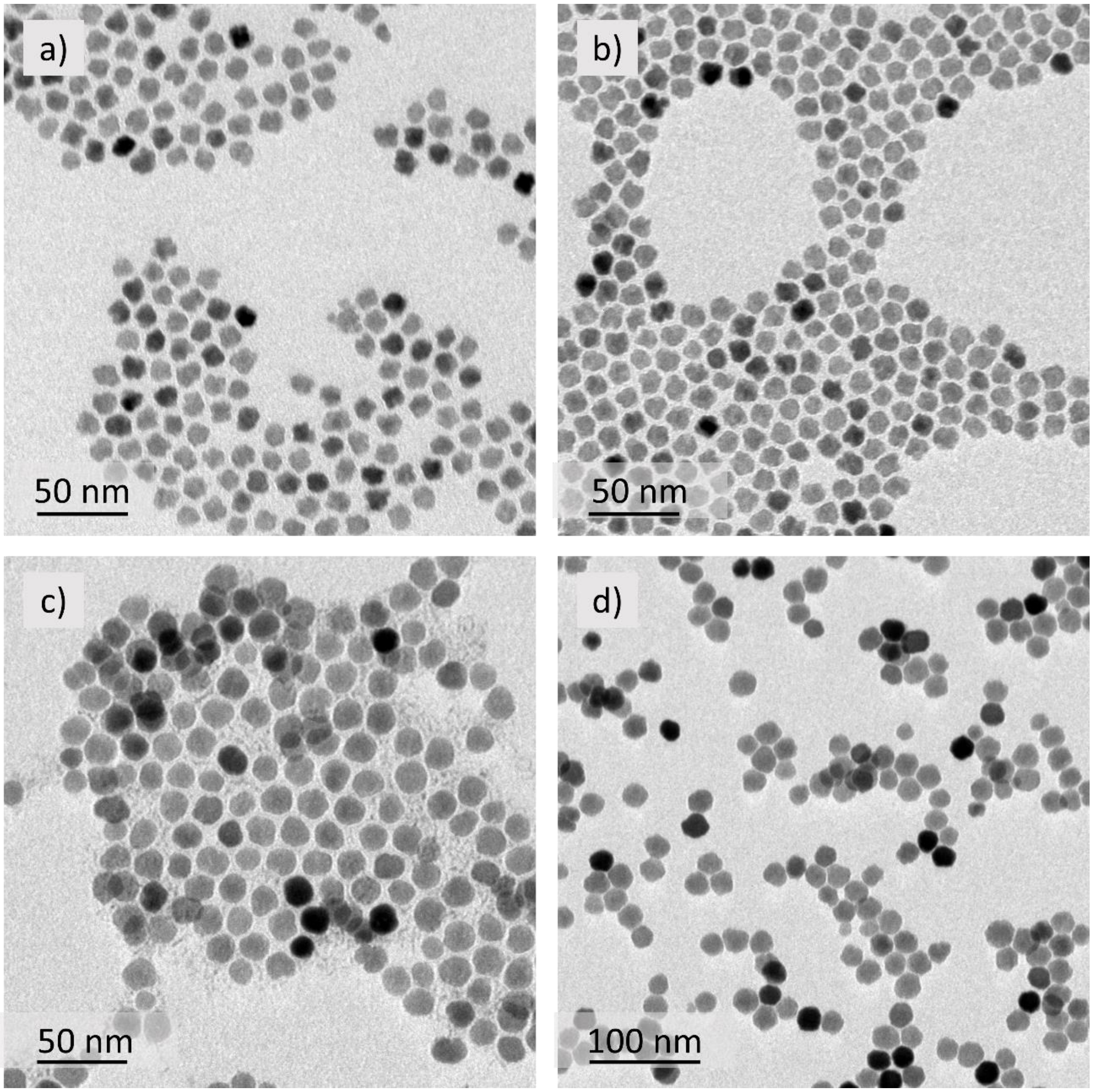

Figure S7 - TEM micrographs of core/shell NCs. a) Core and b) Core/Shell of an A-sized batch. c) Core and d) Core/Shell of a C-sized batch

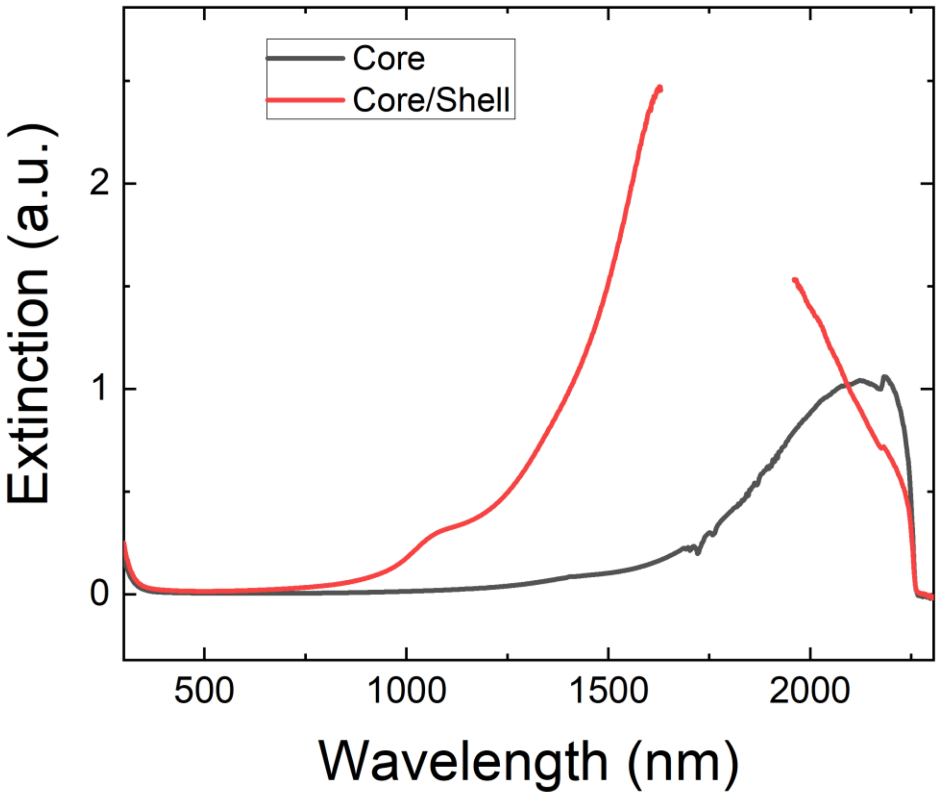

Figure S8 - Extinction spectra of Core and Core/shell structures from a C-sized batch.
